# High-speed broadband absorption spectroscopy enabled by cascaded frequency shifting loops

**DOI:** 10.1038/s41598-023-32763-6

**Published:** 2023-04-08

**Authors:** Hannah M. Ogden, Joseph B. Murray, Matthew J. Murray, Brandon Redding

**Affiliations:** grid.89170.37 0000 0004 0591 0193 U.S. Naval Research Laboratory, 4555 Overlook Ave., SW, Washington, DC 20375 USA

**Keywords:** Engineering, Optics and photonics

## Abstract

Frequency shifting loops, consisting of a fiber optic ring cavity, a frequency modulator, and an amplifier to compensate for loss, enable high-speed frequency scanning with precise and easily controlled frequency steps. This platform is particularly attractive for applications in spectroscopy and optical ranging. However, amplified spontaneous emission noise accumulates due to the repeated amplification of light circulating in the cavity, limiting the frequency scanning range of existing frequency shifting loops (FSLs). Here, we introduce a cascaded approach which addresses this basic limitation. By cascading multiple FSLs in series with different frequency shifts we are able to dramatically increase the accessible scanning range. We present modeling showing the potential for this approach to enable scanning over ranges up to 1 THz—a tenfold increase compared with the state-of-the-art. Experimentally, we constructed a pair of cascaded FSLs capable of scanning a 200 GHz range with 100 MHz steps in 10 ms and used this platform to perform absorption spectroscopy measurements of an H^13^C^14^N cell. By increasing the operating bandwidth of FSLs, the cascaded approach introduced in this work could enable new applications requiring precise and high-speed frequency scanning.

## Introduction

Frequency tunable lasers are essential for a variety of applications including absorption spectroscopy, ranging, LIDAR, and photonic device characterization. While tunable lasers have advanced considerably in recent years^1^, obtaining high-speed frequency tuning with consistent step sizes remains a challenge and many laser scanning systems rely on extensive calibration or in situ monitoring to compensate for non-linearities in the scanned laser frequency^2,3^. An alternative approach is to externally modulate a fixed frequency, continuous wave (CW) laser. However, this approach is typically limited to tuning over modest frequency ranges by the finite bandwidth of optical modulators and the requirement for high-speed drive electronics. Frequency shifting loops (FSLs) provide an attractive alternative by accumulating large frequency shifts by recirculating light through a single modulator 10s or 100s of times^4^.

Frequency shifting loops are typically comprised of a fiber optic ring cavity containing a frequency-shifting modulator, an amplifier which is used to compensate for loss, and a bandpass filter used to suppress amplified spontaneous emission (ASE). After each round-trip in the loop, the light undergoes an additional frequency shift. The FSL can be used to generate an optical frequency comb by seeding it with CW light^5^. Alternately, if pulsed light is coupled into the FSL, it can be used to generate a train of pulses that are equally spaced in time and frequency^6^. This enables precise and high-speed frequency scanning with a relatively low bandwidth modulator and drive electronics. These features have led to the use of FSLs in a wide range of applications including absorption spectroscopy^7–9^, optical frequency comb manipulation^10^, optical Fourier analysis^11^, distributed fiber sensing^12,13^, arbitrary waveform generation^14^, and RF spectrum analysis^15^. The primary drawback of FSLs is that the overall bandwidth is limited by the accumulation of ASE due to the continued amplification of light in the loop. As a result, FSLs are typically limited to a bandwidth of a few 10s of GHz (the broadest band FSL reported to our knowledge spanned 100 GHz^16^) before ASE begins to dominate.

In this work, we introduce a cascaded FSL architecture capable of dramatically increasing the frequency scanning range and the number of frequency steps generated before ASE begins to dominate. We show that combining an initial FSL with smaller frequency steps followed by a second FSL with larger frequency steps allows us to significantly increase the scanning range while minimizing ASE build-up. We present simulations indicating that a properly designed cascaded FSL could enable scanning over 1 THz before ASE begins to dominate. As an initial demonstration, we constructed a cascaded FSL capable of producing 2000 pulses in steps of 100 MHz over a total range of 200 GHz and use the system to perform absorption spectroscopy measurements of an H^13^C^14^N cell. By providing a method to increase the scan range of FSLs, this work will increase the applications for this powerful approach to high-speed, frequency scanning.

## Approach

A frequency shifting loop can provide diverse functionality depending on how it is seeded. For example, if a FSL is seeded with a CW light source, it will generate an optical frequency comb with comb spacing determined by the frequency modulator^5^ and bandwidth dictated by the bandpass filter in the loop. Injecting various modulated waveforms into a FSL provides the potential for arbitrary waveform generation^14^. On the other hand, if the FSL is seeded with an optical pulse, it can be used to generate a train of pulses that are evenly spaced in time and frequency^5^. In this work, we are focused on this last application of FSLs. By generating a pulse train in which each pulse contains a single optical frequency, this approach is particularly well suited for absorption spectroscopy since the absorption at a given frequency can be obtained through direct detection. Compared to conventional tunable laser spectroscopy, FSLs are capable of high-speed scanning while maintaining the high coherence of the seed laser, which could enable applications such as phase-sensitive dispersion spectroscopy. Finally, this approach is amenable to non-linear frequency conversion, enabling access to different frequency regimes^8^.

While this simple approach has enabled high-speed frequency scanning with precisely controlled frequency steps, the total bandwidth and number of pulses generated in the FSL are limited by the accumulation of ASE^17^. The ASE introduced after each round-trip scales with the amplification required to compensate for loss and the bandwidth of the bandpass filter. As a result, mitigating ASE build-up requires the FSL to minimize loss in the loop (reducing the amplification required after each round-trip), to limit the total operating bandwidth (enabling a narrower bandpass filter), and to minimize the number of pulses generated (reducing the number of times the light undergoes amplification). Once the round-trip loss is minimized, ASE build-up effectively introduces a trade-off between the overall bandwidth and the frequency shift between neighboring pulses—a larger bandwidth can be accommodated if the frequency step size is increased to limit the number of pulses generated. In an absorption spectroscopy application, this results in a trade-off between scan range and frequency resolution.

The cascaded FSL approach presented in this work is designed to address this trade-off. Our approach relies on an initial FSL with small frequency shifts, generating a train of pulses that are closely spaced in frequency. These pulses are then used to seed a second FSL which introduces large frequency shifts, exceeding the entire bandwidth of the pulse train generated by the first FSL. This scheme has two advantages. First, using cascaded FSLs reduces the total number of times any individual pulse undergoes amplification. In a conventional, single FSL, the ASE accumulated while generating $N$ pulses scales as $N$. However, in the cascaded scheme, the ASE scales as $2\sqrt{N}$ if $\sqrt{N}$ pulses are generated in each loop. Second, using smaller frequency steps in the initial FSL allows us to use a narrow bandpass filter in the first FSL, further suppressing ASE.

**Figure 1:**
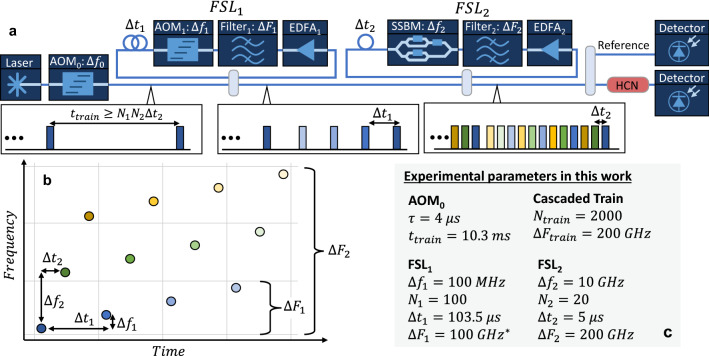
(**a**) Schematic of the cascaded FSL approach. The insets indicate the timing of the pulses generated at each stage. (**b**) Time–frequency diagram indicating the frequency of each pulse, color-coded to match the pulses shown in the insets of (**a**). (**c**) The parameters used in the experimental section to generate a pulse train across 200 GHz with 100 MHz spacing. *Note that $\Delta {F}_{1}=10\text{ GHz}$ would have been optimal, but due to available equipment, a 100 GHz filter was used in the first FSL in the experiments reported in this work.

The basic system architecture is shown in Fig. 1a. The system is seeded with a CW laser and an acousto-optic modulator (AOM_0_) is used to carve an initial seed pulse with pulse duration $\tau$ (note that any intensity modulator could be used in place of AOM_0_). This seed pulse is coupled into the first FSL via a 50:50 coupler. The first FSL consists of an Erbium doped fiber amplifier (EDFA_1_), and bandpass filter with bandwidth $\Delta {F}_{1}$, and a frequency modulator, AOM_1_, which introduces a frequency shift $\Delta {f}_{1}$. The round-trip delay through the first FSL is defined as ${\Delta t}_{1}$. The first FSL generates a train of ${N}_{1}$ pulses separated in frequency by $\Delta {f}_{1}$ and temporally spaced by ${\Delta t}_{1}$, as shown in the inset of Fig. 1a. This initial pulse train then seeds a second FSL consisting of an amplifier, bandpass filter with bandwidth $\Delta {F}_{2}$, and a frequency modulator. Here, an electro-optic single-side band modulator (SSMB) is used to enable relatively large frequency shifts exceeding the bandwidth of the pulse train generated in FSL_1_ (e.g. $\Delta {f}_{2}$ > 1 GHz). The round-trip time in the second loop is defined as $\Delta {t}_{2}$ and should be slightly longer than the pulse duration $\tau$. The number of pulses generated in the second loop, ${N}_{2}$, then sets a limit on the required delay in the first loop as ${\Delta t}_{1}\ge {N}_{2}{\Delta t}_{2}$. Similarly, the delay between the seed pulses and the length of the overall pulse train is ${t}_{train}\ge {N}_{1}{\Delta t}_{1}\ge {N}_{1}\left({N}_{2}{\Delta t}_{2}\right).$ Under these conditions, the output of the second FSL will be a train with ${N}_{1}\cdot {N}_{2}$ total pulses. The pulses do not increase monotonically in frequency, but rather increase in steps of $\Delta {f}_{2}$ before resetting to the frequency of the next pulse out of FSL_1_, as shown in Fig. 1b and color-coded in the inset of Fig. 1a. In principle, it is possible to use smaller delays in the first FSL and longer delays in the second FSL to generate a pulse train that increases monotonically in frequency. However, this would result in uneven delays between pulses arriving at EDFA_1_ in the first FSL and increase the impact of EDFA saturation effects. In practice, we found that the approach shown in Fig. 1, where ${\Delta t}_{1}\gg {\Delta t}_{2}$, enables a stable pulse train with more uniform amplitude in each pulse.

## Modeling

To optimize this scheme, we first simulated the ASE build-up following the model introduced in Ref.^17^. The model was used to calculate the signal-to-noise ratio (SNR) between the signal power in the *n*th pulse, ${P}_{sig}(n)$, and the ASE power in the *n*th pulse, integrated across $m$ frequency bins of width $\Delta f$:

1 $$SNR\left(n\right)= \frac{{P}_{sig}(n)}{\sum_{m}{P}_{ASE}(m,n)}.$$

The signal power in the *n*th pulse was calculated as:

2 $${P}_{sig}\left(n\right)={P}_{sig}\left(n-1\right)\cdot {G}_{EDFA}\left(n\right)\cdot T,$$

where ${G}_{EDFA}\left(n\right)$ is the EDFA gain experienced by the *n*th pulse and $T$ is the transmission through the FSL, including 50% loss due to the fiber coupler. The ASE power in the *m*th frequency bin of width $\Delta f$ after the *n*th round-trip through the FSL was calculated as:

3 $${P}_{ASE}\left(m,n\right)=\left[{G}_{EDFA}\left(n\right)-1\right]\cdot h\nu \cdot NF\cdot\Delta f+\left[{G}_{EDFA}\left(n\right)\cdot T\right]\cdot {P}_{ASE}\left(m-1,n-1\right),$$

where $h$ is Planck’s constant, $\nu$ is the optical frequency, and $NF$ is the noise figure of the EDFA. The first term in this expression accounts for the ASE generated by the EDFA in the *n*th loop while the second term accounts for ASE that continues to circulate in the loop and undergoes a frequency shift $\Delta f$ after each round-trip. The total number of frequency bins was determined by the bandpass filter as $\Delta F/\Delta f$. This approach implies a rectangular bandpass filter that rejects ASE power once it shifts past the edge of the filter.

To account for gain saturation, the EDFA gain experienced in the *n*th round-trip was calculated as

4 $${G}_{EDFA}\left(n\right)={e}^{{g}_{ss}/\left[1+\left[{P}_{tot}(n)/{P}_{sat}\right]\right]},$$

where ${g}_{ss}$ is the small signal gain, ${P}_{sat}$ is the EDFA saturation power, and ${P}_{tot}\left(n\right)$ is the total optical power circulating in the *n*th round-trip, defined as:

5 $${P}_{tot}\left(n\right)={P}_{sig}\left(n\right)+{\sum }_{m}{P}_{ASE}\left(m,n\right).$$

To initialize the simulation, the signal power, ${P}_{sig}(n=1)$, was set to half the power in the seed pulse (to account for the 50% coupler) while the initial ASE power in each frequency bin was set to zero, i.e. ${P}_{ASE}\left(m,n=0\right)=0$. This assumes that the modulator is blocked after the *N*th pulse to reject build-up of ASE from the previous pulse train. In the simulation, the small signal gain was solved for numerically to produce a pulse train with uniform amplitude, insuring that the saturated EDFA gain was approximately equal to the round-trip loss. This is analogous to the experimental procedure of adjusting the EDFA gain to balance loss in the FSL and generate a uniform pulse train.

To evaluate the cascaded approach shown in Fig. 1, we first used the model described above to calculate the signal and ASE power produced by the first loop. The simulated signal and ASE powers were then provided as inputs to the second FSL and we calculated the SNR of the final pulse train leaving the second FSL.

**Figure 2:**
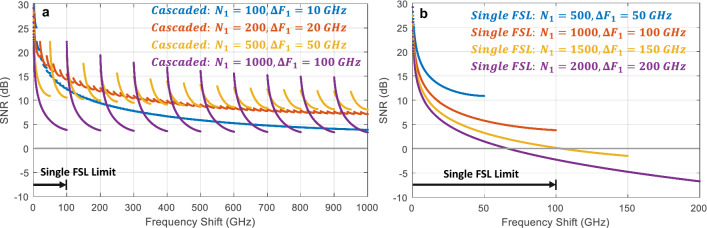
(**a**) Simulated SNR vs frequency shift for a cascaded pulse train covering 1 THz with 100 MHz steps. The number of pulses generated in the first FSL was varied from 100 to 1000. The cascaded approach enables 10,000 pulses over 1 THz with SNR > 7 dB. (**b**) Simulated SNR for a single FSL designed to generate pulse trains with 100 MHz spacing across 50 to 200 GHz. The single FSL can only generate a pulse train covering ~ 100 GHz with SNR > 0 dB.

To illustrate the advantage of the cascaded approach, we simulated a system designed to produce 10,000 pulses covering 1 THz in steps of 100 MHz. This is significantly beyond the reach of a single FSL (100 GHz is the widest bandwidth reported using a single FSL^16^). In this case, we assumed transmission through both loops of 0.1 (i.e. 10 dB of loss) and a 50% coupler, such that the total round-trip transmission was ${T}_{1}={T}_{2} =0.05$. We then varied the number of pulses generated in the first FSL from ${N}_{1}=$ 10 to 100 and set the bandwidth of the filter in the first loop to $\Delta {F}_{1}={N}_{1}\Delta {f}_{1}$ with a fixed frequency shift of $\Delta {f}_{1}=100 {\text{ MHz}}$. The frequency shift in the second loop was set to $\Delta {f}_{2}={N}_{1}\Delta {f}_{1}$ with a fixed bandpass filter bandwidth of $\Delta {F}_{2}=1 \,{\text{THz}}$ in order to support the entire pulse train. As shown in Fig. 2a, it is possible to maintain an SNR > 7 dB across 10,000 pulses by using the first FSL to generate 200 pulses covering 20 GHz. This shows the potential for this approach to dramatically extend the operating range of FSLs. The number of pulses generated in each FSL should be optimized based on the overall bandwidth of the desired pulse train and the loss in each loop. In this case, the SNR is considerably lower if the first FSL was used to generate 100 pulses or 1000 pulses rather than the ideal 200 to 500 pulses. For comparison, we also modeled the SNR of a single FSL with the same loss ($T=0.05$) designed to produce pulse trains covering 50 to 200 GHz in steps of 100 MHz. In each case, the bandpass filter was set equal to the total bandwidth of the generated pulse train. As shown in Fig. 2b, the single FSL cannot provide frequency shifts exceeding ~ 100 GHz before the SNR drops below 0 dB. In general, the acceptable SNR will depend on the application and this type of model can be used to study the bandwidth which can be achieved using a cascaded FSL while maintaining a required SNR.

**Figure 3:**
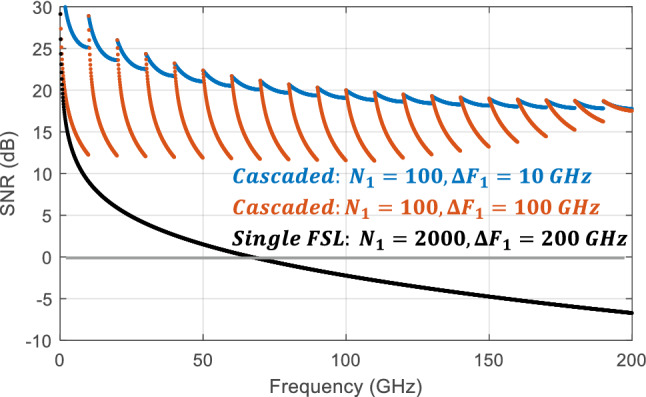
Simulated SNR vs frequency shift for a cascaded pulse train using the experimental conditions in this work. This pulse train was designed to cover 200 GHz in 100 MHz steps using the first FSL to generate 100 pulses over 10 GHz. The simulation includes the experimental case using a 100 GHz bandpass filter as well as the optimal case using a 10 GHz bandpass filter. In both cases, the cascaded FSL approach is able to maintain an SNR > 10 dB across the entire span. The SNR if a single FSL was used to generate a 200 GHz pulse train is also shown, indicating that the SNR drops below 0 dB after ~ 60 GHz.

Finally, we adjusted the model parameters to match our experimental parameters, providing the SNR plot shown in Fig. 3. Experimentally, we designed the cascaded FSLs to generate 2000 pulses covering 200 GHz in steps of 100 MHz. The first FSL was used to generate 100 pulses over 10 GHz, while the second FSL provided 20 pulses in steps of 10 GHz. Due to constraints on available equipment, our experimental system exhibited higher loss than the system simulated in Fig. 2a and relied on a broader bandpass filter in the first FSL than would have been ideal. In this simulation, we matched our experimental conditions, using the measured loss values of ${T}_{1}=0.05$ and ${T}_{2}=0.0005$. The high loss in the second FSL was caused by low transmission through the SSBM due to the lack of an available RF signal generator with enough power to reach ${V}_{\pi }$. We also used a 100 GHz bandpass filter in the first loop in the experiment, rather than the 10 GHz filter which would have been optimal. Figure 3 shows the SNR we would have expected with the ideal 10 GHz filter along with the SNR expected using a 100 GHz filter. While the 10 GHz filter clearly provides superior performance, both designs enable an SNR > 10 dB across the entire 200 GHz. Curiously, the SNR actually improves at the end of the pulse train using the 100 GHz filter. This was due to ASE generated in the first loop (covering a 100 GHz band) which was eventually shifted outside of the bandpass filter in the second FSL near the end of the pulse train. For comparison, we also modeled the SNR we could expect if we tried to use the first FSL to cover the entire 200 GHz range. In this case, we used the same loss of ${T}_{1}=0.005$ and the bandpass filter was set to ${\Delta F}_{1}=200\text{ GHz}.$ As shown in Fig. 3, the SNR drops below 0 dB after only ~ 60 GHz, clearly showing the advantage of the cascaded approach.

## Experimental demonstration

We constructed a cascaded FSL following the basic architecture shown in Fig. 1a, starting with a narrowband seed laser (Rio Orion) operating at an optical frequency of $\nu =193.53\text{ THz}$ (wavelength of $\lambda =1549.1\text{ nm}$ in vacuum) with output power of 10 mW and linewidth $<1$ kHz. We then used 100 MHz AOMs (Brimrose AMMF-100) to carve the initial seed pulse and introduce the frequency shift in the first FSL. A 100 GHz wavelength division multiplexing filter (AFW Technologies, WDM-PM) was used in the first FSL to suppress ASE. In the second FSL, a single sideband modulator (SSBM, Thorlabs LN86S-FC) was used to introduce the frequency shift and an additional 100 MHz AOM was included to reject ASE light between pulse trains (a 10 GHz frequency shift in the second FSL was provided by driving the SSBM at 9.9 GHz and the AOM at 100 MHz). A tunable filter (Santec OTF-980) was used to position a 200 GHz bandpass filter starting at the seed laser frequency. The entire system was constructed using single mode fiber, polarization maintaining fiber couplers (Thorlabs PN1550R5A2), and benchtop EDFAs (Thorlabs EDFA100s). As described above, the system was designed to generate 2000 pulses across 200 GHz in steps of 100 MHz by generating 100 pulses in the first FSL and 20 pulses in the second FSL. The experimental parameters are summarized in Fig. 1c.

**Figure 4:**
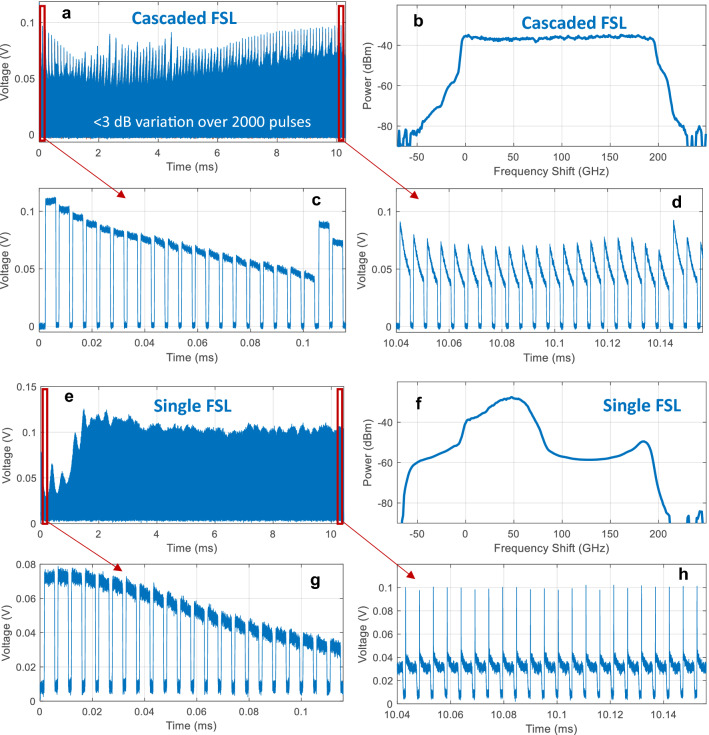
(**a**) Pulse train generated by the cascaded FSL containing 2000 pulses. (**b**) Measured spectrum produced by the cascaded pulse train. The cascaded pulse train produced the expected flat spectrum across 200 GHz with a relatively weak pedestal due to ASE leaking through at the edge of the bandpass filter. (**c**) Magnified view of the first 22 pulses generated by the cascaded FSL and (**d**) 22 pulses near the end of the pulse train. (**e**) Pulse train generated by a single FSL containing 2000 pulses. (**f**) A measured spectrum produced by a single FSL. A distorted spectrum is produced due to the significant ASE build-up. (**g**) Magnified view of the first 22 pulses generated by a single FSL and (**h**) 22 pulses near the end of the pulse train.

The pulse train recorded on the reference photodetector (Terahertz Technologies, TIA-525) shown in Fig. 1a is presented in Fig. 4a. The pulse train reveals 2000 evenly spaced, 4 μs pulses across 10.3 ms. The pulses near the beginning of the pulse train, shown in Fig. 4c, are relatively rectangular while the pulses at the end of the pulse train, shown in Fig. 4d, exhibit some distortion due to EDFA saturation effects^18^. The EDFA introduces a very slight distortion each time a pulse is amplified, which accumulates in the loop, resulting in a more noticeable distortion in the pulses shown in Fig. 4d which have undergone nearly 200 amplification events. Nonetheless, the overall spectrum, recorded on an optical spectrum analyzer with wavelength resolution $\Delta \lambda =0.08\text{ nm} (\Delta \nu =10\text{ GHz})$, confirms that the pulse train spanned the desired 200 GHz with less than 3 dB of amplitude variation, as shown in Fig. 4b. For comparison, the spectrum produced when a single FSL was used to generate 2000 pulses across the same 200 GHz is shown in Fig. 4f. In this case, the spectrum is severely distorted due to the build-up of ASE. The pulse train measured using a single FSL is also shown in Fig. 4e,g,h. Similar to the cascaded FSL, the pulses near the beginning of the pulse train generated in a single FSL are fairly rectangular, as shown in Fig. 4g. However, the accumulation of ASE in the single FSL setup causes significant distortion to the pulses near the end of the pulse train, which were amplified as many as 2000 times, as shown in Fig. 4h. The rapid accumulation of ASE in a single FSL also introduces significant fluctuations in the overall power level, as shown in Fig. 4e.

**Figure 5:**
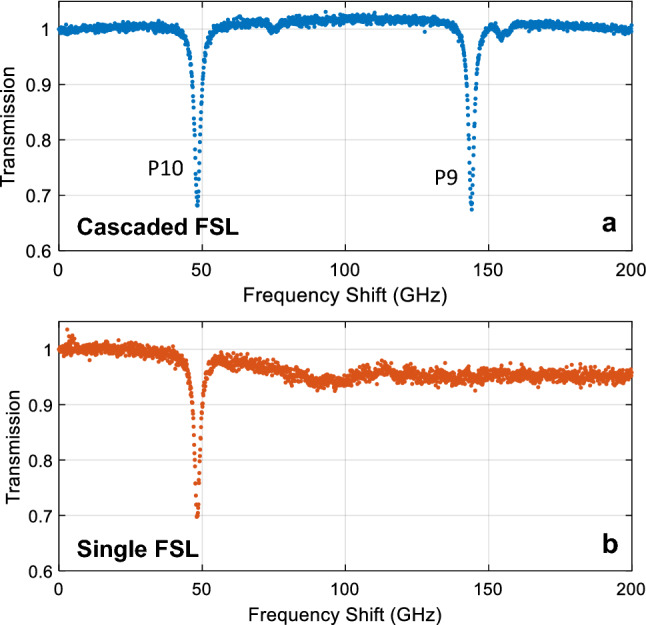
(**a**) H^13^C^14^N absorption spectrum recorded using the cascaded FSL correctly revealing the presence of the P10 and P9 absorption lines. (**b**) The absorption spectrum recorded using a pulse train generated with a single FSL is only able to record the first absorption line—ASE build up dominates at higher frequencies using a single FSL, precluding an accurate absorption measurement.

We then used the pulse train generated by the cascaded FSLs to perform an absorption spectroscopy measurement by probing a fiber-coupled gas cell containing H^13^C^14^N (Wavelength References, HCN-13-H-(5.5)-25-FCAPC) with a pressure of 25 Torr and a path length of 5.5 cm. In our experiment, light passed twice through the cell (using a fiber-coupled Faraday mirror and a polarizing beamsplitter), providing an effective absorption pathlength of 11 cm. The absorption spectrum was measured by recording the power of each pulse on the two detectors shown in Fig. 1a. We then calculated the average power during the center 2 μs of each pulse (to minimize the impact of the EDFA saturation effects shown in Fig. 4d) and calculated the transmission as the relative power between the reference pulse and the pulse which passed through the H^13^C^14^N cell. The absorption spectrum obtained using the cascaded pulse is shown in Fig. 5a, revealing 2 ro-vibrational lines separated by 96 GHz, as expected^19^. The full width at half maximum of the absorption lines was ~ $2.4\text{ GHz}$, which agrees with previous spectroscopic measurements of the $2{\nu }_{3}$ transition of H^13^C^14^N at room temperature and 25 Torr^19^. This linewidth includes contribution from both Doppler broadening ($450\text{ MHz})$ and pressure broadening ($2.2\text{ GHz})$^19^. Note that this measurement was obtained in 10.3 ms—the length of one pulse train, without requiring additional averaging. The standard deviation in transmission measured at the end of the pulse train (i.e. in the last 10 GHz) was 0.0036.

Although the ratio of the tone to ASE background level is expected to vary across the pulse train (as shown in Fig. 3), this variation is not directly observable in the transmission spectrum. Instead, the modest background ASE level (expected to comprise less than 10% of the power in each pulse based on the modeling shown in Fig. 3) primarily impacts the modulation depth of the absorption line. In contrast, the impact of ASE is much more obvious in Fig. 5b, which shows the transmission spectrum recorded using a single FSL. In this case, only the first absorption line was observed. This is consistent with the simulation shown in Fig. 3, which indicated that the single FSL would only maintain an SNR > 0 dB up to a shift of ~ 60 GHz. Since the pulses that should have probed the second absorption line near 140 GHz were dominated by broadband ASE, no absorption was observed. This confirms that the cascaded FSL approach can enable spectroscopy measurements over a larger bandwidth than a single FSL.

## Conclusions

We introduced a scheme based on cascaded frequency shifting loops to increase the frequency scanning bandwidth of FSLs. This approach has two advantages: it reduces the number of amplification events experienced by any individual pulse and enables a much lower bandwidth filter in the first FSL—both of which reduce ASE build-up. The simulations presented in this work indicate that a cascaded FSL with relatively low loss (10 dB) could enable scanning over 1 THz. Experimentally, we showed that even with relatively high insertion loss components, this approach enables scanning over 200 GHz in 100 MHz steps. By increasing the scanning range of FSLs, this cascaded approach could enable additional applications for this convenient approach to high-speed, high-resolution frequency scanning.

## Data Availability

The data generated during this study are available from the corresponding author upon reasonable request.
